# Non-neutralizing Antibodies Directed at Conservative Influenza Antigens

**DOI:** 10.32607/20758251-2019-11-4-22-32

**Published:** 2019

**Authors:** E. S. Sedova, D. N. Scherbinin, A. A. Lysenko, S. V. Alekseeva, E. A. Artemova, M. M. Shmarov

**Affiliations:** Federal Research Centre for Epidemiology and Microbiology named after the honorary academician N.F. Gamaleya of the Ministry of Health of the Russian Federation, Moscow, 123098 Russia

**Keywords:** influenza virus, broad-spectrum influenza vaccine, antibody-dependent cellular cytotoxicity, antibody-dependent cellular phagocytosis, antibody-mediated complement-dependent cytotoxicity

## Abstract

At the moment, developing new broad-spectrum influenza vaccines which would help avoid annual changes in a vaccine’s strain set is urgency. In addition, developing new vaccines based on highly conserved influenza virus proteins could allow us to better prepare for potential pandemics and significantly reduce the damage they cause. Evaluation of the humoral response to vaccine administration is a key aspect of the characterization of the effectiveness of influenza vaccines. In the development of new broad-spectrum influenza vaccines, it is important to study the mechanisms of action of various antibodies, including non-neutralizing ones, as well as to be in the possession of methods for quantifying these antibodies after immunization with new vaccines against influenza. In this review, we focused on the mechanisms of anti-influenza action of non-neutralizing antibodies, such as antibody-dependent cellular cytotoxicity (ADCC), antibody-dependent cellular phagocytosis (ADCP), and antibody-mediated complement-dependent cytotoxicity (CDC). The influenza virus antigens that trigger these reactions are hemagglutinin (HA) and neuraminidase (NA), as well as highly conserved antigens, such as M2 (ion channel), M1 (matrix protein), and NP (nucleoprotein). In addition, the mechanisms of action and methods for detecting antibodies to neuraminidase (NA) and to the stem domain of hemagglutinin (HA) of the influenza virus are considered.

## INTRODUCTION

Influenza is a highly contagious infection; it is responsible for annual epidemics and periodical pandemics that appear at varied intervals. According to the WHO, 20–30% of children and 5 to 10% of adults are infected with influenza annually in the world and 250 to 500 thousand people die from severe complications of the influenza infection. In pandemics, the extent of complications and mortality increase significantly. For instance, according to various sources, around 50 to 100 million people died from influenza during the 1918–1919 flu pandemic [1].

The most potent protective measure against the influenza infection and its spread is vaccination. Modern influenza vaccines, as a rule, induce the formation of antibodies to the influenza HA and NA surface antigens. The surface proteins of the influenza virus undergo constant antigenic drift. Therefore, annual renewal of the strain composition of the vaccine is required [2].

To date, the development of new broad-spectrum influenza vaccines which would help avoid the necessity of annual changes in the strain composition of the vaccine remains urgency. In addition, the creation of new vaccines based on highly conserved influenza virus proteins would allow us to better prepare for potential pandemics and significantly reduce the damage they cause.

**Fig. 1:**
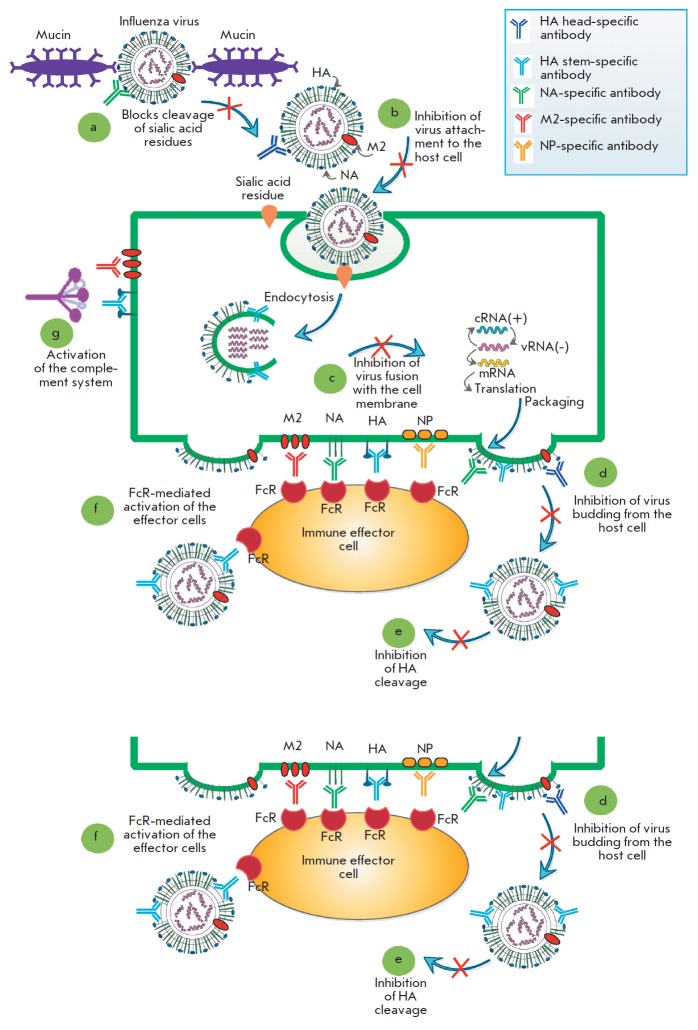
Mechanisms of action of anti-influenza antibodies. The influenza virus enters the body through respiratory tract mucosa, where viral hemagglutinin (HA) binds to the terminal sialic acids of mucin. Neuraminidase (NA) releases the virus by cleaving the terminal sialic acid residues. Antibodies to neuraminidase can inhibit the reaction, and the virus would not be able to penetrate the mucous layer **(a)**. After penetrating the mucous layer, the influenza virus binds to the sialic acids on the surface of the target cells and enters the cell by endocytosis. Neutralizing antibodies bind to influenza HA and block this process **(b).**The endosomes of the target cells become acidified, thus triggering the fusion of the endosomal and viral membranes via HA, which results in the release of the viral genome into the cell cytoplasm. Antibodies to the stem domain of HA can inhibit this process **(c).**After the synthesis of viral proteins, the internal proteins are packed into viral particles containing HA, NA, and the M2 ion channel molecules on the virion surface. On the cell surface, the HA, NA, and M2 proteins can be bound by antibodies that block the budding of viral particles. Maturing viral particles are covered by the host cell membrane as a result of the interaction between HA and sialic acids. Meanwhile, NA cleaves terminal sialic acids from the virus, while antibodies to NA can inhibit this process **(d)**. Finally, in the matured viral particles, HA0 is cleaved into the HA1 and HA2 subunits by the host proteases that are present in the respiratory tract. Antibodies directed to the HA stem domain can block this process **(e).**In addition, viral antigens exposed to the surface of an infected cell (including the internal protein NP, which is detected on the surface of the infected cell) are targets for antibodies that activate effector cells via the Fc-FcR interaction **(f).**Antibodies directed to the viral antigens exposed on the cell surface can also activate the complement system **(g) **

The key to evaluating the effectiveness of influenza vaccines is to determine the level of humoral response after vaccination. Neutralizing antibodies to the globular head domain of hemagglutinin are produced during viral infection and undergird the protective mechanisms of all the influenza vaccines available to date [3]. Most virus-neutralizing antibodies bind to the head domain of HA, inhibit the binding of HA to the sialic acid residue and prevent the virus from entering the cells *(Fig. 1, b)*. These antibodies are determined by conventional hemagglutination inhibition and neutralization reactions [4-6]. Moreover, many HA head-specific antibodies are also able to inhibit the release of the virus from the cell *(Fig. 1, d)*. This defense mechanism cannot be evaluated by conventional hemagglutination inhibition and neutralization inhibition assays; it is detected by adding antibodies to cells that have been previously infected with the influenza virus [7].

Antibodies against various conserved antigens of the influenza virus (such as NP, M1, M2) are generally non-neutralizing in nature and cannot prevent the development of the viral infection. However, they are able to exert a protective function through various immune mechanisms. Thus, the study of the mechanisms of action of various antibodies, including non-neutralizing ones, as well as the development of methods for evaluating the level of such antibodies after immunization with new influenza vaccines, is relevant for the development of novel broad-spectrum influenza vaccines.

## ANTIBODIES TO CONSERVED ANTIGENS OF THE INFLUENZA VIRUS PARTICIPATING IN THE REACTIONS OF ANTIBODY-DEPENDENT CELLULAR CYTOTOXICITY, ANTIBODY-DEPENDENT PHAGOCYTOSIS, AND ANTIBODY-MEDIATED COMPLEMENT-DEPENDENT CYTOTOXICITY

The ability of antibodies to neutralize the influenza virus has traditionally been considered the most important mechanism of protection against influenza. However, recent studies have shown the importance of other antibody-mediated effects, which also contribute to antiviral protection [3]. The following mechanisms of anti-influenza action are realized by non-neutralizing antibodies: antibody-dependent cellular cytotoxicity (ADCC), antibody-dependent cellular phagocytosis (ADCP), and antibody-mediated complement-dependent cytotoxicity (CDC) [8]. The influenza virus antigens that trigger these reactions are HA, NA, and highly conserved antigens such as the M2 ion channel, M1 matrix protein, and nucleoprotein (NP).

Unlike neutralizing antibodies, the functions of which are implemented by the variable regions, the effect of non-neutralizing antibodies depends on the conserved Fc region. The Fc region is able to interact with various components of the immune system, while the variable part of the antibody binds to the antigen. The most significant antibody isotypes for the implementation of the effector functions of non-neutralizing antibodies are IgG and IgM, with IgG3 possessing the highest functional potential [9].

Neutralizing antibodies can bind with their Fc region to the specific Fc receptors exposed on the surface of most immune cells, including NK cells, macrophages, and neutrophils *(Fig. 1, f)*. After binding to antibodies, these immune cells are activated and become involved in the defense response against a pathogen. A total of six different receptors involved in the activation (FcγRI, IIA, IIC, IIIA, and IIIB) or inhibition (FcγRIIB1/B2) of human immune cells have been described. Non-neutralizing antibodies can also activate the complement system *(Fig. 1, g)* [9].

**Antibody-dependent cellular cytotoxicity (ADCC)**

**Fig. 2:**
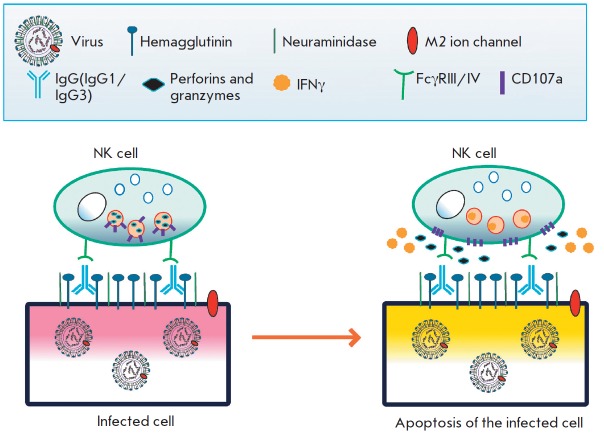
The mechanism of antibody-dependent cellular cytotoxicity (ADCC) in a cell infected with an influenza virus. IgG binds to the viral antigens on the surface of the infected cell. NK cells recognize the infected cells via Fc-FcR interactions and then release cytotoxic granules and secrete antiviral cytokines

Influenza virus-infected cells carry viral proteins on their surface – mainly HA and NA – since new virions are formed by budding from the cell membrane. Anti-influenza IgG can bind viral proteins on the cell surface, thus opsonizing the infected cells. The Fc gamma receptor IIIa (FcγRIIIa) exposed on the surface of many cells of the innate immune response, such as NK cells, monocytes, and macrophages, binds to the Fc region of IgG. The interaction between FcγRIIIa and IgG bound to the infected cell leads to phosphorylation of the tyrosine-based activation motif (ITAM) and activation of the Ca^2+^-dependent signaling pathway. As a result, NK cells begin to produce cytotoxic factors (perforins and granzymes), which lead to the death of the infected cell, and antiviral cytokines (IFNγ, TNFα) and chemokines *(Fig. 2)* [10].

One of the main targets for the antibodies involved in ADCC is the conserved stem of HA, which is one of the most represented surface proteins of the influenza virus. For instance, it has been shown that antibodies with a broad spectrum of activity against the conserved HA stem protect mice from a lethal influenza infection through a mechanism that involves an interaction with Fc-FcγR. On the contrary, the protective activity of antibodies in relation to the variable head domain of HA has manifested itself both in the presence and absence of an interaction with FcγR [11].

Furthermore, re-infection of macaques with the influenza virus has led to a rapid appearance of ADCC responses. Antibodies capable of inducing activation of NK cells were found in the bronchoalveolar lavages of the macaques, which correlated with a reduced virus shedding and decreased disease duration [12]. In humans, high titers of antibodies capable of participating in ADCC were also shown to correlate with a decrease in the incidence of an experimental infection [13]. Moreover, elderly people who had previously been infected with viruses close to the strain that caused the 2009 swine influenza pandemic and who retained a significant amount of titers of the antibodies participating in ADCC but had no neutralizing antibodies were protected from the pandemic influenza virus. Thus, non-neutralizing HA stem-specific antibodies capable of inducing ADCC are directly related to the level of protection against an influenza virus [14]. In addition, according to published data, vaccines against seasonal influenza viruses weakly induce the production of antibodies that can participate in ADCC, while the presence of NK cell-activating antibodies with a broad spectrum of activity in elderly people suggests that these antibodies accumulate over a lifetime as a result of re-infection with various influenza virus strains [15].

Using a panel of 13 monoclonal antibodies to the influenza virus HA protein (both neutralizing and non-neutralizing ones, both stem- and head-specific ones), DiLillo et al. [16] showed that Fc-FcγR interactions are necessary for all broad-spectrum antibodies in order to ensure *in vivo *protection. A similar result was obtained by comparing two NA-specific antibodies, one of which had a broad spectrum of action; the other was strain-specific. This suggests that the spectrum of action of not only certain HA-specific antibodies, but also antibodies to other influenza antigens exposed on the surface of an infected cell, depends on the Fc-FcγR interaction. Moreover, the dependence of some antibodies on the Fc-FcγR interaction can be circumvented by significantly (8–10-fold) increasing the amount of the antibody involved in the interaction with the influenza virus. It should be noted that, during viral infection, broad-spectrum antibodies are generated in much smaller quantities than strain-specific ones. Thus, the Fc-FcγR interaction apparently can increase the efficiency of the broad-spectrum antibodies, thereby compensating for their small quantity [16].

Antibodies to conserved viral proteins, such as nucleoprotein (NP), also contribute to the ADCC response. For instance, the influenza infection and vaccination induce the production of antibodies to the NP, M1, and M2 proteins involved in ADCC [17, 18]. It has been shown that influenza virus NP is expressed on the surface of infected cells for some time and, therefore, can serve as a target for ADCC [19-21]. Carragher et al. demonstrated that vaccination of laboratory mice with soluble recombinant NP of the influenza A virus induces high titers of antibodies to NP and an extremely weak T cell response. At the same time, vaccination reduced the manifestation of disease symptoms and decreased the influenza virus titers in the lungs of the influenza-infected animals infected. Passive transfer of the sera of immunized mice to naive animals also provided protection against an influenza infection [22]. Subsequent studies have demonstrated that the protective effects of the serum of mice immunized with influenza A recombinant NP upon passive transfer to animals with B cell deficiency and mice with a normal number of B cells manifest themselves through the mechanism that includes FcγR [23]. Macaque studies have shown that NP-specific antibodies have the ability to activate NK cells *in vitro *[18, 24].

The serum of healthy children and adults (but not infants) contains antibodies to various proteins of the H7N9 influenza A virus that are involved in the ADCC response, with the level of NP-specific antibodies being significantly higher than those to HA and NA. The level of antibodies to NP of the seasonal influenza A viruses that are involved in ADCC correlated with the level of antibodies to NP of the H7N9 influenza A virus. Therefore, production of these antibodies that cross-react with H7N9 is assumed to be triggered by vaccination and infection with seasonal influenza A viruses [25]. The antibodies to influenza A virus NP involved in ADCC were found in children vaccinated with seasonal inactivated influenza virus vaccines. NP-specific antibodies that can interact with FcγRIIIa and activate NK cells have been identified in healthy and influenza-infected volunteers. Healthy donor serum containing NP-specific antibodies were shown to induce NK cell activation against virus-infected cells expressing NP [13, 26].

Another conserved influenza protein found on the surface of infected cells is the M2 ion channel. Antibodies to this protein can protect mice from an influenza virus infection in laboratory experiments. Moreover, in the immunization of animals with the M2 ectodomain both in soluble form and as conjugated to various carriers, the protective ability depends mainly on the antibodies. Notably, the presence of NK cells was critical to protection. [27]. Experiments on passive immunization of both wild-type mice and mice with the FcRγ-/-, FcγRI-/-, FcγRIII-/-, and (FcγRI, FcγRIII)-/- phenotypes showed that FcR (more specifically, FcγRIII) is required for the protective effect of anti-M2e antibodies [28, 29]. The human monoclonal antibody against the influenza A (Ab1-10) virus M2 protein was able to activate NK cells and trigger ADCC *in vitro*, with ADCC against both target cells expressing M2 and cells infected with influenza [30].

**Fig. 3:**
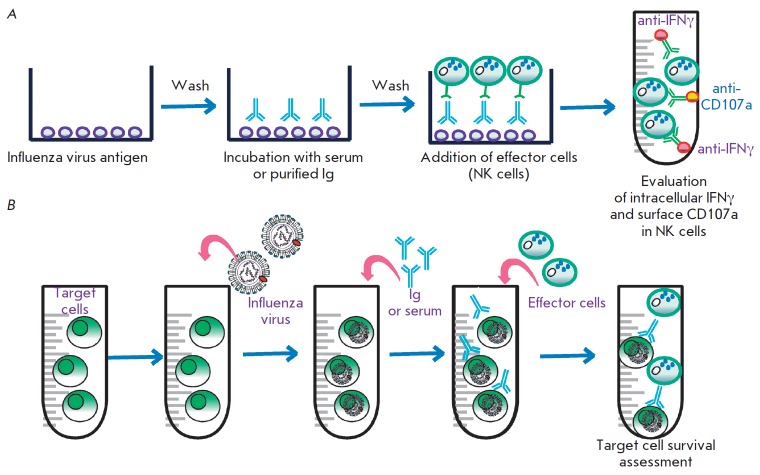
Evaluation of ADCC in the laboratory. (a) Evaluation of ADCC using immobilized influenza virus antigens. Firstly, antigens are immobilized, washed, and incubated with serum or IgG isolated from the blood. Secondly, unbound IgGs are washed off and effector cells (peripheral blood monocytes or isolated NK cells) are added to the antigen-antibody complex. Thirdly, after incubation, activation of the effector cells is analyzed. Activation is evaluated by adding labeled antibodies to the surface and secreted marker proteins (as a rule, the surface membrane activation marker CD107a and interferon gamma are used). (b) Evaluation of ADCC using influenza virus-infected cells or target cells expressing the major viral antigens. Cells expressing viral antigens are incubated with serum or preliminarily purified IgG. Next, effector cells are added to the antibody-treated cells and ADCC is evaluated by counting dead target antibody-treated cells

To accurately determine the level of anti-influenza antibodies involved in ADCC, a reaction with the participation of the target antigen and effector cells (usually NK cells) is required. The antigen can be either a recombinant target protein, influenza-infected cells, or target cells expressing the desired antigens. If the antigen is a recombinant protein, it is treated with the test serum and then effector cells are added to the resulting antigen–antibody complex. When conducting this reaction, one can assess ADCC by measuring the activation of the effector cells and their expression of surface and secreted marker proteins (as a rule, these are surface activation marker CD107a and interferon gamma) *(Fig. 3, a)* [31]. If the antigen is infected cells or target cells, ADCC can also be analyzed by assessing the death rate of antibody-treated target cells after their interaction with effector cells *(Fig. 3, b)* [32].

**Antibody-dependent cellular phagocytosis (ADCP)**

**Fig. 4:**
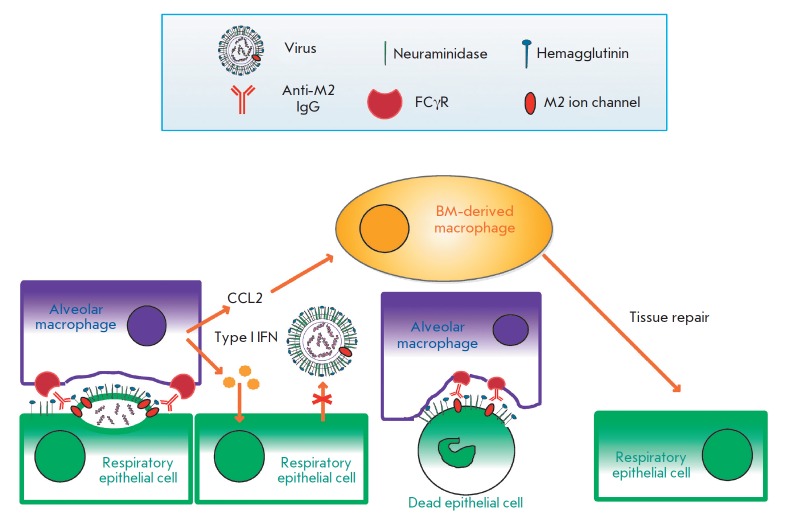
The mechanism of the protective action of the antibodies binding to the ectodomain of the influenza M2 ion channel. Respiratory tract epithelial cells infected with the influenza A virus expose the HA, NA, and M2 viral proteins on their surface. New viral particles are budding from the infected cells. On the surface of the budding virion, antibodies bind to the ectodomain of the M2 protein and opsonize the viral particle. These antibodies activate alveolar macrophages via Fcγ receptors. Activated macrophages are able to phagocytize budding virions and fragments of the cell membrane containing the M2 protein. Dying infected cells can be also opsonized by antibodies to the M2 ectodomain and phagocytosed by alveolar macrophages via the FcγR-dependent pathway. Activated macrophages also produce type I interferons, which possess antiviral activity and regulate the expression of chemokine CCL2, which attracts bone marrow macrophages promoting tissue repair

Phagocytosis is a crucial immunological process in which phagocytes engulf microbial and infected cells. The first step of ADCP includes opsonization of a microbial or infected cell by antibodies. After opsonization, phagocytes recognize the antibodies bound to foreign antigens, mainly via the Fcγ receptors CD32 (FcγRIIA) and CD64 (FcγRIA), as well as the Fcα receptor CD89 [33]. The phagocytes involved in ADCP include monocytes, macrophages, neutrophils, and dendritic cells *(Fig. 4)* [17, 34]. ADCP is one of the most important antibody-induced effector defense mechanisms against the influenza virus. FcγR-/- mice have been shown to be highly sensitive to influenza even in the presence of influenza antibodies obtained from FcγR+/+ mice. Moreover, the absence of NK cells was not crucial for the defense response. It has also been shown that FcγR+/+ mouse macrophages actively engulf opsonized viral particles [35]. Dunand et al. showed that some non-neutralizing human broad-spectrum monoclonal antibodies protect mice from an influenza infection through Fc-mediated recruitment of effector cells, with the protection being associated exclusively with ADCP but not with ADCC or activation of the complement system [36].

According to He et al., alveolar macrophages are crucial for the induction of ADCP by human and mouse monoclonal antibodies both *in vitro *and in experiments on protecting animals from infection with homologous and heterologous influenza A virus strains [37]. Interestingly, the ability of alveolar macrophages to protect the lungs from damage during an influenza infection is reduced in elderly mice [38].

In addition to alveolar macrophages, other effector cell populations can also participate in the ADCP-mediated response to the influenza virus. For instance, neutrophils, which are the largest in number amongst blood leukocytes, express high levels of FcγRIa/b/c, FcγRIIa, and FcγRIIIb on their surface after activation. In addition, neutrophils constitutively express FcαRI, which binds IgA and activates the cytotoxic and phagocytic responses [15]. Analysis of the Fc-FcγR interactions between various IgG specific to the HA stem and effector neutrophils showed that monoclonal human and mouse HA stem-specific antibodies can induce the production of reactive oxygen species (ROS), which are further delivered to the neutrophil`s phagolysosomes. However, such an effect could not be detected in the case of HA head-specific antibodies [39]. The depletion of neutrophils resulted in a reduced survival rate of influenza-infected mice [40, 41].

A study of the ADCP mechanism in the influenza infection showed that both macrophages and neutrophils are quickly recruited to the lungs and are present in bronchoalveolar lavage, the respiratory tract, and alveoli, where they contribute to the rapid scavenging of infected and dead cells. Although the supernatant of influenza-infected cells can stimulate phagocytosis by monocytes regardless of the involvement of antibodies [40], antibodies contribute to the effective clearance of viral particles and infected cells by interacting with the FcγRIa and FcγRIIa on immune cells. Antibody-mediated viral phagocytosis causes a decrease in the infection spread and severity, as well as it symptoms, and a reduction in virus shedding [42]. It is assumed that each subsequent influenza infection, as well as influenza vaccinations, slightly induces the cross-reactive antibodies involved in ADCP, with their level increasing with each subsequent influenza infection [17].

Various anti-influenza antibodies, including antibodies to the hemagglutinin stem [36, 37, 39] and antibodies to the M2 ion channel [28, 43], can induce ADCP.

The activity of the antibodies responsible for ADCP is studied as follows: target cells expressing influenza antigens are labeled with an intravital dye, then the target antibodies and phagocytic cells are added, and the survival number of the target cells is assessed. [35].

**Antibody-mediated complement-dependent cytotoxicity (CDC)**

The complement system consists of soluble and membrane-bound proteins that are found in the blood and tissues of mammals. These proteins interact with each other and with other components of the immune system, resulting in the production of a number of effector proteins that contribute to the elimination of various pathogens [27].

As early as in 1978, it was shown that the complement system is necessary to protect mice from a lethal influenza infection [44]. In 1983, it was established that human serum contains antibodies capable of neutralizing the influenza virus by activating the classical complement pathway [45]. To date it is known that influenza virions can activate both the classical and alternative complement pathways, and that antibody opsonization is required for efficient lysis of virions [46].

In 2018, research was carried out to study the effectiveness of immunization of mice with a knockout of the C3 complement component with virus-like particles carrying the M2e proteins of human, porcine, and avian influenza A viruses, as well as virus-like particles carrying HA of the H5-subtype influenza A virus. It turned out that immunization with the M2e vaccine did not protect C3 knockout mice from the influenza A viruses, while even low levels of antibodies to the M2e protein were enough to protect wild-type animals from the influenza A virus infection upon passive transfer. On the contrary, C3 knockout mice immunized with a HA vaccine, which induces the production of strain-specific neutralizing antibodies, were protected from infection with a homologous influenza virus despite the low level of antibody response [47]. Thus, one can state the ability of antibodies to the influenza virus M2 ion channel to protect against influenza A virus infection through the activation of the complement system.

The complement system is not only capable of neutralizing viral particles, but it is also involved in the lysis of infected cells. For instance, vaccination with a seasonal trivalent inactivated influenza vaccine led to an increase in the level of antibodies capable of activating the complement-dependent lysis of influenza-infected cells *in vitro*, although the effect was not pronounced [48]. CDC-inducing antibodies were detected among both influenza HA head-specific and stem-specific antibodies. Meanwhile, antibodies to the stem domain demonstrated a broad spectrum of action and were able to induce CDC against different influenza A strains [49].

Antibodies of the IgG1 and IgM classes have been shown to be involved in the activation of the complement system [46]. The level of antibodies involved in CDC correlated with the protection against a seasonal influenza virus in children [32].

The activity of antibodies in CDC is evaluated by the rate of death of target cells expressing the influenza antigen or infected with an influenza virus in solutions containing complement components and antibodies. Cell death is assessed using various metabolic dyes [50].

## ANTIBODIES TO INFLUENZA VIRUS HEMAGGLUTININ AND NEURAMINIDASE

Influenza virus HA and NA are highly variable proteins. However, the broad-spectrum vaccines that are currently under development may also include HA and/or NA and their epitopes. For instance, the HA stem is a rather conserved part of the molecule. Various strategies exist for redirection of the immune response to this particular antigen during vaccination [51]. Most NA-specific monoclonal antibodies obtained from the serum of people who have been infected bind to different influenza strains, providing ground for the development of broad-spectrum vaccines based on this antigen [52].

**Antibodies to the influenza virus HA stem**

The HA stem is more conserved than the head domain. However, it is less immunogenic, possibly due to the fact that the bulky head region of the protein sterically hinders the access of antibodies to the HA stem. However, in addition to the non-neutralizing antibodies involved in the ADCC, ADCP, and CDC reactions, a certain amount of neutralizing HA stem-specific antibodies is detected in humans after an influenza infection or vaccination, with the infection being more effective in inducing the formation of this type of antibodies than vaccination.

HA stem-specific antibodies can interfere with the fusion of the virus with the endosomal membrane. Effective fusion requires the presence of 3–5 neighboring HAs with fusion peptides bound to the endosomal membrane *(Fig. 1, c)*. Neutralizing HA stem-specific are able to prevent the pH-induced exposure of the fusion peptide and prevent the formation of a network of HAs interacting with the endosomal membrane. In addition, some antibodies to the HA stem can inhibit cleavage of the immature HA0 precursor into the HA1 and HA2 subunits *(Fig. 1, e)*, which are required for a successful infection of cells with a newly formed viral particle [4]. HA stem-specific antibodies can suppress the release of viral particles from the cell *(Fig. 1, d)* [53], including the pathway involving steric inhibition of neuraminidase activity [54].

Neutralizing HA stem-specific antibodies cannot be revealed using a hemagglutination assay. The principal methods for an evaluation of these antibodies are the reactions of neutralization [5], microneutralization [6], and neutralization based on pseudotyped viral vectors [55]. The latter present chimeric viruses carrying influenza virus surface antigens that do not contain any genetic material and are not infectious. These pseudoviruses are usually derived from lentiviral vectors and the vesicular stomatitis virus. They allow avoidance of highly pathogenic influenza strains when performing a neutralization reaction (which is especially important when studying broad-spectrum antibodies). According to some reports, this method of evaluation is more sensitive and more suitable in the detection of neutralizing HA stem-specific antibodies than the conventional neutralization assay [55].

**Antibodies to influenza virus neuraminidase**

Influenza virus neuraminidase is involved in various stages of the infectious process. It cleaves the viral particles from the sialic acid residues of the respiratory tract mucins, thus allowing for the virus entry into the cell. NA allows the release of new virions from the host cell, thus preventing them from remaining bound to the sialic acid residues on the cell surface. In addition, NA prevents the aggregation of virions, which is due to the interaction between the HA of a virion with the sialylated glycans of another one [56]. Antibodies to the influenza virus NA can interfere with any of these processes *(Fig. 1, a,d)*.

It was shown that, in the absence of HA-specific antibodies, NA-specific antibodies can protect laboratory animals from an influenza infection [57]. Moreover, the presence of NA-specific antibodies also correlates with protection against an influenza virus in humans. The titer of anti-NA antibody has been shown to increase in human blood with age [56].

The production of antibodies to neuraminidase is induced by an influenza infection. However, their level is usually lower than that of the antibodies to HA. It is crucial that most NA-specific monoclonal antibodies derived from the serum of individuals who have suffered from an infection bind to a wide range of modern and historical influenza strains, inhibit NA activity, and protect laboratory mice in passive transfer experiments [52]. Several strategies for the development of broad-spectrum vaccines that induce the formation of antibodies to influenza NA exist. One of them is the creation of a pandemic vaccine based on a cocktail of several subtypes of NA (N1, N2, N6, N7, N8, N9, and etc.) associated with human and zoonotic influenza strains. It is also possible to include NA as an additional antigen in vaccines based on conserved influenza antigens: such as M2, NP, etc. [58].

ELISA is one of the easiest ways to evaluate the immune response to NA. To reliably assess the level of NA-specific antibodies, recombinant, tetrameric, glycosylated, and enzymatically active NA should be used as antigen. However, ELISA does not provide any information on the functionality of the measured level of antibodies. NA enzymatic activity inhibition assays are based on the cleavage of small molecules by neuraminidase, which generates the signal to be measured. However, unlike terminal sialic acids, which are attached to the glycans of large proteins, these small molecules more easily access the active center of the NA protein [59]. The assay that allows one to obtain the most realistic estimates of the anti-NA activity of antibodies is ELLA (enzyme-linked lectin assay), which uses the highly sialylated glycoprotein fetuin as a substrate. The method is based on measuring the amount of galactose, the penultimate sugar residue in fetuin, which is bound to the substrate. NA cleaves terminal sialic acids, after which galactose can be measured using horseradish peroxidase-conjugated peanut lectin. ELLA has been optimized for routine serology; it is now used to evaluate the titers of neuraminidase-inhibiting antibodies [58].

A key component of ELLA is enzymatically active NA, the inhibition of which is evaluated. NA can be used in the form of a purified protein or as part of a viral particle. When using a viral particle, it should be kept in mind that HA-binding antibodies can reduce NA activity due to the steric hindrance effect. Therefore, reassortant viruses of the H6NX and H7NX subtypes are commonly used in this assay. Although the use of reassortant viruses cannot completely exclude the effect of HA-specific antibodies on the NA activity, this assay is considered as the "gold" standard in the evaluation of the inhibitory anti-NA activity of antibodies [59].

## CONCLUSIONS

In this review, the main mechanisms involving anti-influenza antibodies and the methods for the detection of these antibodies were considered. Antibodies can provide protection against influenza via Fc-independent or Fc-dependent mechanisms. Fc-independent antibodies directly neutralize the virus by preventing its entry into the cell, fusion, or budding from it. Antibodies to the head domain of hemagglutinin [5], which are usually strain-specific, are mainly involved in the direct neutralization of the influenza virus. As a rule, Fc-dependent antibodies are non-neutralizing but are able to activate antibody-dependent cellular cytotoxicity, antibody-dependent cellular phagocytosis, or the complement system [18, 34, 49]. Such antibodies can be targeted at the stem domain of the HA, NA, M2, or NP proteins of the influenza virus, and most of them are broad-spectrum antibodies [11, 21, 28, 58].

The influenza vaccines currently being developed are aimed at generating an immune response not mainly to the conventional HA and NA influenza antigens, but to various conserved viral antigens. When creating a broad-spectrum vaccine, it is necessary to know what effectiveness criteria should be considered in preclinical and clinical trials. The traditional methods for assessing the humoral immune response to influenza vaccines by hemagglutination and neutralization reactions will no longer be relevant for most newly developed vaccines. The development of methods for evaluating non-neutralizing anti-influenza antibodies and studying their mechanisms of action are necessary if we seek to create effective broad-spectrum vaccines that can provide protection against both seasonal and potentially pandemic influenza virus strains.

## Abbreviations

ADCC: antibody-dependent cellular cytotoxicity
ADCP: antibody-dependent cellular phagocytosis
CDC: antibody-mediated complement-dependent cytotoxicity
HA: hemagglutinin
NA: neuraminidase
NP: nucleoprotein
WHO: World Health Organization
DNA: deoxyribonucleic acid
NK: natural killers
ITAM: immunoreceptor tyrosine-based activation motif
ELISA: enzyme-linked immunosorbent assay
IFNγ: interferon gamma
TNFα: tumor necrosis factor alfa
ELLA: enzyme-linked lectin assay
